# Gallium nitride nanowire as a linker of molybdenum sulfides and silicon for photoelectrocatalytic water splitting

**DOI:** 10.1038/s41467-018-06140-1

**Published:** 2018-09-21

**Authors:** Baowen Zhou, Xianghua Kong, Srinivas Vanka, Sheng Chu, Pegah Ghamari, Yichen Wang, Nick Pant, Ishiang Shih, Hong Guo, Zetian Mi

**Affiliations:** 1 Department of Electrical and Computer Engineering, McGill University, 3480 University Street, Montreal, QC H3A 0E9 Canada; 2 Department of Physics, McGill University, 3600 University Street, Montreal, QC H3A 2T8 Canada; 3 Department of Electrical Engineering and Computer Science, Center for Photonics and Multiscale Nanomaterials, University of Michigan, 1301 Beal Avenue, Ann Arbor, MI 48109 USA

## Abstract

The combination of earth-abundant catalysts and semiconductors, for example, molybdenum sulfides and planar silicon, presents a promising avenue for the large-scale conversion of solar energy to hydrogen. The inferior interface between molybdenum sulfides and planar silicon, however, severely suppresses charge carrier extraction, thus limiting the performance. Here, we demonstrate that defect-free gallium nitride nanowire is ideally used as a linker of planar silicon and molybdenum sulfides to produce a high-quality shell-core heterostructure. Theoretical calculations revealed that the unique electronic interaction and the excellent geometric-matching structure between gallium nitride and molybdenum sulfides enabled an ideal electron-migration channel for high charge carrier extraction efficiency, leading to outstanding performance. A benchmarking current density of 40 ± 1 mA cm^−2^ at 0 V vs. reversible hydrogen electrode, the highest value ever reported for a planar silicon electrode without noble metals, and a large onset potential of +0.4 V were achieved under standard one-sun illumination.

## Abstract (web summary)

Sunlight-harvesting materials require the clean integration of light-absorbing and catalytic components to be efficient. Here, authors link silicon photoelectrodes and molybdenum sulfide catalysts with defect-free gallium nitride nanowire to improve photoelectrochemical hydrogen evolution.

## Introduction

Hydrogen generation via photoelectrochemical (PEC) water splitting is an appealing approach for the conversion of solar energy into chemical fuel^1,2^. At the heart of a PEC cell is the photoelectrode. An ideal photoelectrode, which composes of the photoabsorber for harvesting solar light and catalyst for reducing protons, should be cost-effective, absorb a large part of the solar spectrum, and have an efficient catalyst for improving the kinetics of the hydrogen evolution reaction (HER)^3^. What is more, an ideal electron-migration channel between photoabsorber and catalyst for high charge carrier extraction efficiency is in urgent demand. Over the past decades, many material systems have been extensively studied, including Si^4–7^, metal oxides^8^, III–V semiconductors^9,10^, and others^11^. For metal oxides, they generally suffer from inefficient solar light absorption and limited charge carrier extraction due to their large bandgap (>2.0 eV) and the low mobility and short lifetime of charge carriers^12^. High performance devices have been obtained by III–V compounds but at high cost and complexity^13^. In contrast with metal oxides and III–V semiconductors, silicon is earth-abundant and has a suitable bandgap (1.1 eV) for absorbing a large portion of the solar spectrum. It is worth noting that planar silicon (Si) has been well developed and widely used in photovoltaic industry, thus being one of the most attractive candidates for photoelectrodes^14^. The HER kinetics of bare silicon, however, is extremely sluggish^15^. A suitable and inexpensive catalyst, coupling planar silicon with an efficient electron-migration channel, is thus required.

Molybdenum sulfides (MoS_*x*_), which have received tremendous attention in recent years^16,17^, are considered as a promising catalyst to accelerate the kinetics of planar silicon because of its superior HER catalytic activity and low cost^18–20^. Thus far, much effort has been devoted to the direct decoration of planar silicon with molybdenum sulfides for solar water splitting and great progress has been made^21,22^. However, the utilization of conventional MoS_*x*_/planar Si as photocathodes for achieving high performance still remains a grand challenge, due to the inefficient solar light harvesting of planar Si related to the strong scattering of light^23^, and limited surface area of planar Si leading to a low density of exposed active sites^24^. Most importantly, it is of difficulty in realizing efficient charge carrier extraction between MoS_*x*_ and planar Si for high solar-to-hydrogen efficiency because of the interfacial defects, chemical incompatibility, and synthesis difficulties^25^. It would be of particular interest to seek for an efficient linker between MoS_*x*_ and planar Si to overcome these critical challenges, especially to improve the interfacial and electronic properties of MoS_*x*_/planar Si for providing an efficient electron-migration channel.

Metal-nitrides, for instance, gallium nitride (GaN), have emerged as a new generation of materials for solar water splitting due to its unique structural, electrical, and optical properties^26^. The recent development of molecular beam epitaxy leads to controlled synthesis of single-crystal GaN nanowire arrays on planar Si with a high-quality interface and dramatically reduced manufacturing cost^27^. These as-grown GaN nanowire arrays possess defect-free structure and large charge carrier mobility, resulting in efficient charge carrier extraction from Si substrate^28^. Furthermore, the structure of nanowire arrays is beneficial for exposing high-density active sites and enhancing solar light absorption^29,30^.

Herein, we investigate the use of defect-free GaN nanowires as an ideal linker between planar Si wafer and molybdenum sulfides catalyst. Using density functional theory calculations, we discover that, due to the unique electronic interaction and excellent geometric-matching structure between GaN and MoS_*x*_, the interface of MoS_*x*_/GaN is highly favorable for charge carrier extraction. Experimentally, we demonstrate super shell-core heterostructure of MoS_*x*_@GaN NWs/Si by combining electrodeposition of molybdenum sulfides with molecular beam epitaxy of GaN nanowires. The integrated photocathode is completely noble-metal-free, and exhibits high catalytic activity and stability for PEC water splitting. A benchmarking current density of 40 ± 1 mA cm^−2^ at 0 V vs. RHE (all the potentials in this work are referenced to RHE), as well as a high onset potential of +0.4 V is achieved under standard one-sun illumination. Our unique approach of constructing super heterostructures offers tremendous benefits for solar water splitting, including the use of low-cost, large-area Si wafer for light harvesting, earth-abundant MoS_*x*_ catalyst for proton reduction, and defect-free GaN nanowires for highly efficient charge carrier extraction and for exposing high-density active sites. It further provides a promising direction for achieving low-cost, high-efficiency artificial photosynthesis through the integration of multiscale and multifunctional materials.

## Results

### Calculated atomic structures

**Fig. 1:**
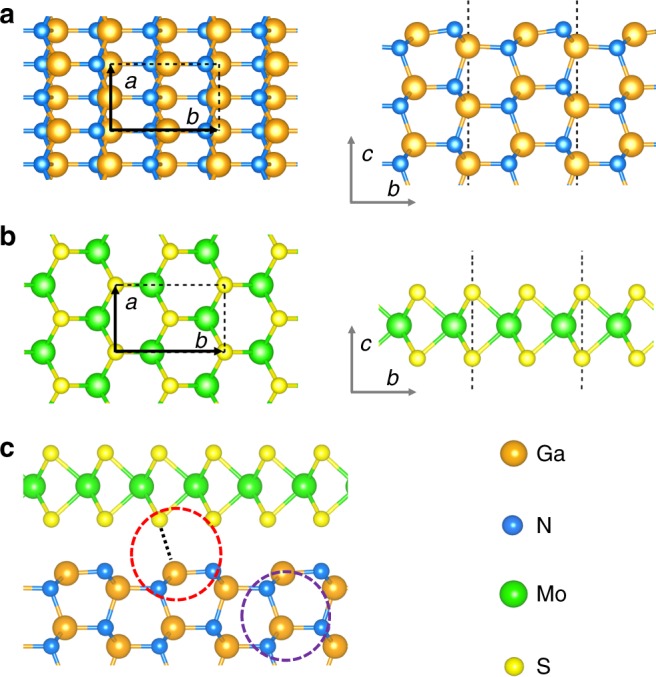
Calculated atomic structures. Top and side view of the fully relaxed atomic structures of **a** GaN (10$\bar 1$0)-wurtzite and **b** MoS_2_. **c** Top view of the fully relaxed atomic structures of MoS_2_/GaN (10$\bar 1$0) vertical heterointerface

Shown in Fig. 1 are the fully relaxed atomic structures of GaN (10$\bar 1$0)-wurtzite, MoS_2_ (1 L) and their corresponding vertical heterointerface. The relaxed lattice parameters for the primitive cell of bulk-GaN and MoS_2_ are 3.198 Å (Fig. 1a) and 3.161 Å (Fig. 1b), respectively, which are in excellent accordance with their experimental values, i.e., 3.186 Å^31^, and 3.160 Å^32^. These two materials have nearly perfect geometric matching, wherein the lattice mismatch is just 0.8%. As is marked in the red and purple circle in Fig. 1c, S atoms prefer to sit above Ga atoms, and form the same spatial arrangement with Ga atoms as the N atoms in the bulk region. Moreover, the GaN dimer formed from surface reconstruction becomes nearly flat in the heterointerface. The unique geometry information of GaN (10$\bar 1$0)-wurtzite and MoS_2_ provides a strong theoretical support that an excellent MoS_2_/GaN (10$\bar 1$0)-wurtzite heterointerface can be constructed^33,34^, exhibiting great potential for efficient charge carrier extraction for water splitting, which has never been investigated before.

### Schematic illustration and characterization of photocathode

**Fig. 2:**
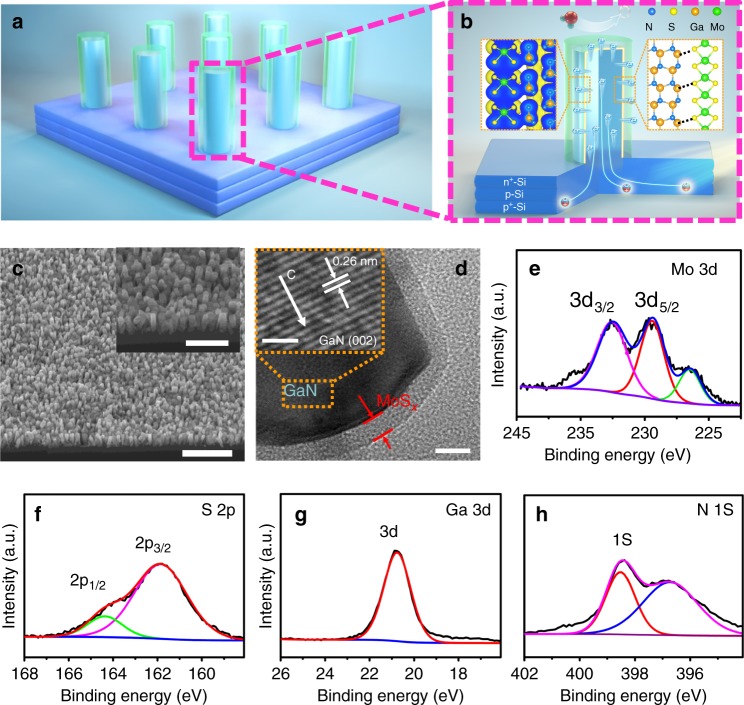
Schematic illustration and structural characterization. **a**, **b** Schematic illustration of the MoS_*x*_@GaN NWs/Si heterostructure. GaN nanowire core covered with a uniform shell of MoS_*x*_ (light-green section) is vertically aligned on planar n^+^–p junction silicon (the left inset of **b** shows the unique electronic interaction of MoS_2_/GaN while the right part signifies the outstanding geometric matching between MoS_2_ and GaN). **c** 45^o^-tilted SEM image of MoS_*x*_@GaN NWs/Si. **d** TEM image of MoS_*x*_@GaN nanowire. Inset of **d** is the HR-TEM image of the GaN core of MoS_*x*_@GaN nanowire. High-resolution XPS spectral of **e** Mo 3d, **f** S 2p, **g** Ga 3d, and **h** N 1s, respectively. Scale bars: **c** 1 μm, inset of **c** 500 nm, **d** 10 nm, inset of **d** 2 nm

In this work, we combined planar Si with MoS_*x*_ using GaN nanowire to develop an outstanding photocathode through a facile two-step process of molecular beam epitaxy and electrodeposition (see Supplementary Figure 1 and Methods for details). The design of MoS_*x*_@GaN NWs/Si photocathode is schematically illustrated in Fig. 2a, b. In such a structure, the n^+^–p junction Si can absorb a large portion of the solar spectrum up to wavelengths of 1100 nm due to its narrow bandgap (~1.1 eV). The incorporation of GaN nanowire arrays enhanced the light harvesting of n^+^–p Si junction, which was confirmed by the UV–Vis reflectance spectra analysis. The relative reflectance spectra indicated that the GaN nanowire arrays improved the light absorption of n^+^–p Si junction in a wide wavelength range (~220–1100 nm) due to the anti-reflection effect^29,30^ (Supplementary Figure 2). What is more, MoS_*x*_@GaN NWs/Si exhibited a further improvement on light absorption compared to GaN NWs/Si. Significantly, the unique electronic interaction and excellent geometric-matching structure between GaN and MoS_*x*_ provides a near-perfect electron-migration channel for high charge carrier extraction efficiency. These synergetic effects lead to photocathode with outstanding performance.

The structure and composites were characterized by scanning electron microscopy (SEM), transmission electron microscopy (TEM), X-ray photoelectron spectroscopy (XPS), and X-ray diffraction (XRD). The SEM image of GaN NWs/Si in Supplementary Figure 3 illustrates that GaN nanowire arrays are vertically aligned on the planar Si substrate with relatively uniform lengths of ~150 nm and diameters varying from 30 to 40 nm. After electrodeposition, the GaN nanowires are covered with MoS_*x*_ and the morphology of nanowire arrays are well retained (Fig. 2c). The TEM characterization in Fig. 2d demonstrates that an evident shell-core heterostructure of MoS_*x*_@GaN nanowires is formed. The thickness of the MoS_*x*_ nanolayer is about 7 nm. HR-TEM further illustrates that the GaN core of shell-core MoS_*x*_@GaN nanowire shows almost defect-free crystalline structure; and the lattice spacing of 0.26 nm between the two adjacent (002) plane suggests the growth direction along the *c*-axis (Inset of Fig. 2d)^35^. The binding energies of the elements in MoS_*x*_@GaN NWs/Si were characterized by XPS (Supplementary Figure 4). The high-resolution Mo 3d spectrum in Fig. 2e shows two peaks at 232.9 eV (Mo 3d3/2) and 229.6 eV (Mo 3d5/2), indicating that Mo^4+^ is the dominant oxidation state; the typical peaks of S 2p at 161.5 eV (S 2p3/2) and 163.5 eV (S 2p1/2) are assigned to S^2−^ (Fig. 2f)^36^. The binding energies of Ga 3d (20.8 eV) and N 1s (398.1 eV) confirm the presence of GaN (Fig. 2g, h). X-ray diffraction was further performed to examine the as-synthesized sample (Supplementary Figure 5). It is discovered that the typical peak of GaN at 2*θ* = 34.5°, which is ascribed to the (002) plane, appears at both GaN NWs/Si and MoS_*x*_@GaN NWs/Si^35^. The diffraction peaks of MoS_*x*_ are however not observed. This result suggests that the electrodeposited MoS_*x*_ is likely amorphous, which is in agreement with the TEM examination and previous report^37^. The amorphous phase of MoS_*x*_ facilitates H_2_ evolution owing to the high degree of structural disorder and more catalytic active sites^38^, even though there may be certain amount of dangling bonds in the interface, which is worthy of further investigation.

### Photoelectrochemical measurements

**Fig. 3:**
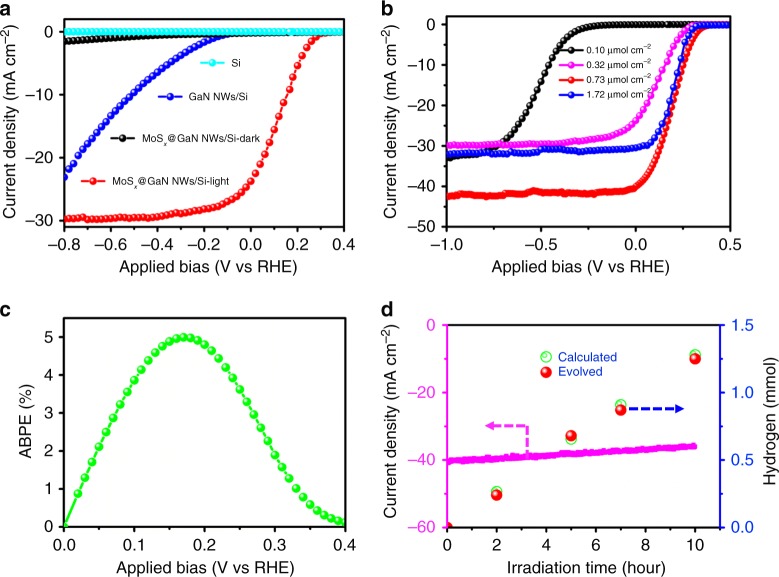
PEC water splitting performance in 0.5 M H_2_SO_4_ under standard one-sun illumination. **a** *J*–*E* curves of various photocathodes of planar n^+^–p junction Si, GaN NWs/Si, and MoS_*x*_@GaN NWs/Si. **b** *J*–*E* curves of MoS_*x*_@GaN NWs/Si with different loading densities of MoS_*x*_. **c** ABPE of MoS_*x*_@GaN NWs/Si with 0.73 μmol cm^−2^ of MoS_*x*_ versus applied potential. **d** Stability and Faradaic efficiency measurements of MoS_*x*_@GaN NWs/Si

Linear sweep voltammetry (LSV) measurements were conducted to study the PEC water splitting performance of bare planar n^+^–p junction Si, GaN NWs/Si, and MoS_*x*_@GaN NWs/Si in 0.5 M H_2_SO_4_ under standard one-sun illumination (100 mW cm^−2^) using a three-electrode configuration (Fig. 3). As illustrated in Fig. 3a, the photocurrent density of pristine planar n^+^–p junction Si is essentially negligible even at a highly negative potential of −1.0 V vs. RHE. In contrast, the performance of GaN NWs/Si is remarkably enhanced; and the onset potential, corresponding to a photocurrent density of −0.2 mA cm^−2^ is at −0.06 V vs. RHE. The improvement could be attributed to both enhanced light absorption and more efficient extraction of electrons from Si substrate by GaN nanowires. However, photocurrent is still not observed at 0 V. Strikingly, after depositing 0.32 μmol cm^−2^ of MoS_*x*_, MoS_*x*_@GaN NWs/Si shows a substantial improvement in both onset potential as well as photocurrent density compared to those of bare Si and GaN NWs/Si. A current density of 24.7 mA cm^−2^ at 0 V with an onset potential of +0.32 V is obtained under standard one-sun illumination. It is seen that MoS_*x*_@GaN NWs/Si exhibits negligible activity in the dark, due to the absence of the photogenerated charge carriers, indicating that solar energy is the driving force for the reaction. The influence of the loading density of MoS_*x*_ on the reaction was studied (Fig. 3b). It is discovered that the catalytic activity gradually improves with increasing the loading density of MoS_*x*_ from 0.10 to 0.73 μmol cm^−2^. Further loading the catalyst to 1.72 μmol cm^−2^, however, leads to a reduced performance. The main reason is that the loading density of MoS_*x*_ affects the reaction in opposite ways. In the catalytic cycle, MoS_*x*_ serves as a catalyst and provides active sites for the hydrogen evolution reaction. On the other hand, excessive loading of MoS_*x*_ would result in the block of more incident light^39^. What is more, the density of MoS_*x*_ evidently affects the morphology of MoS_*x*_@GaN NWs. From Supplementary Figure 6, it is found that the thickness of MoS_*x*_ increases with loading density. Compared to the uniform MoS_*x*_/GaN NWs shell-core structure at loading density of 0.73 μmol cm^−2^, a higher density of 1.72 μmol cm^−2^ produces a thicker and irregular MoS_*x*_ layer, which exhibits a decreased activity. The interplay among these factors renders 0.73 μmol cm^−2^ to be an optimal value, allowing for an extraordinary balance of high-quality shell-core heterostructure, sufficient active sites, and efficient solar light absorption. The variation of the optimized photocathode’s performance is tested and illustrated in Supplementary Figure 7, which is found to be small. Under this situation, a benchmarking current density of 40 ± 1 mA cm^−2^ at 0 V and an onset potential as high as +0.4 V are achieved. To our best knowledge, this value is the highest ever reported for planar Si photocathodes in the absence of noble metals (Summary in Table 1). As a result, a maximum applied bias photon-to-current efficiency (ABPE) of 5.0% is achieved at +0.16 V (Fig. 3c). In addition, the length of GaN NWs affected the activity obviously. As illustrated in Supplementary Figure 8, when the GaN NW length increased from 150 to 250 nm, both the onset potential and the current density of the device decreased.

**Table 1:** The summary of planar silicon with non-noble-metal catalysts for water splitting

Photocathodes	Electrolyte	Onset potential (V vs. HRE)	Current density at 0 V vs. RHE (mA cm^−2^)	Refs.
MoS_*x*_@GaN NWs/Si	0.5 M H_2_SO_4_	+0.40	−40 ± 1	This work
NiCoSe_*x*_/planar p-Si	0.5 M H_2_SO_4_	+0.07	−5.1	4
WC_2_/planar p-Si	1 M H_2_SO_4_	+0.2	−5	5
CoP/planar n^+^p Si	0.5 M H_2_SO_4_	+0.46	−20	6
Mo_3_S_4_/planar Si	1.0 M HClO_4_	+0.15	−9	7
a-CoMoS_*x*_/planar Si	H_3_PO_4_	+0.25	−17.5	16
MoS_2_/planar n^+^p Si	0.5 M H_2_SO_4_	+0.32	−17	17
MoS_*x*_/Ti/planar n^+^p Si	1 M HClO_4_	+0.33	−16	21
n-MoS_2_/planar p-Si	0.5 M H_2_SO_4_	+0.17	−24.6	22
MoS_*x*_Cl_*y*_/planar Si	0.5 M H_2_SO_4_	+0.27	−20.6	25
MoS_2_Cl/graphene/planar Si	0.5 M H_2_SO_4_	+0.27	−20.6	38
MoS_*x*_/planar p-Si	0.5 M H_2_SO_4_	+0.25	−17	39

Stability and faradaic efficiency measurements were performed using chronoamperometry at a fixed potential of 0 V vs. RHE under standard one-sun illumination (Fig. 3d). There was no observable decrease in photocurrent density after at least 10-h illumination. This indicates a high level of stability of the photoelectrode, which is confirmed by XPS (Supplementary Figure 9a) characterization before and after the reaction. The good stability of the photocathode is mainly attributed to the MoS_*x*_@GaN nanowire arrays, which due to their superior stability that provides a protection layer to the photocathode^40,41^. The high-resolution XPS spectra at the range of Pt 4f did not show typical Pt peaks, excluding that the loss of activity resulted from the redeposition of Pt on the photocathode (Supplementary Figure 9b). Through SEM measurements (Supplementary Figure 10), it is also noticed that some nanowires fell off the Si substrate during the stability test, which could account for the slight loss of the activity. Gas chromatography analysis confirmed that the gaseous product evolved from the photocathode was H_2_. Moreover, the evolved amount of H_2_ is nearly equal to that calculated by the consumed electrons, indicating that the faradaic efficiency is nearly 100%.

### Calculated electronic properties of MoS_2_/GaN (10${\bar{\bf{1}}}$0)

**Fig. 4:**
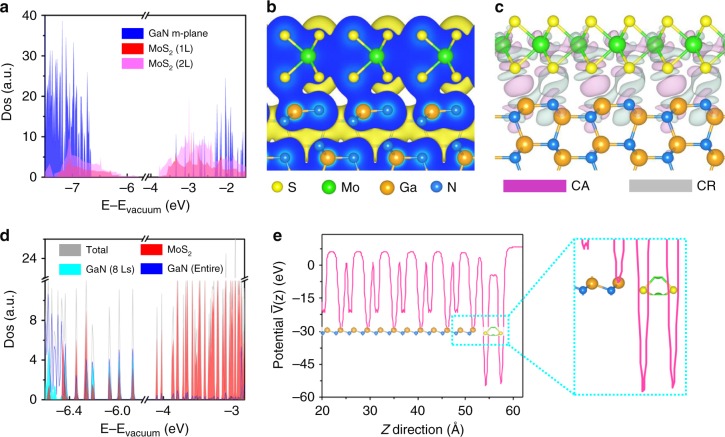
Calculated electronic properties of MoS_2_ /GaN(10$\bar 1$0). **a** Density of states of GaN(10$\bar 1$0), MoS_2_ (1L), and MoS_2_ (2L), respectively. **b** Charge density, **c** differential charge density, and **d** density of states of MoS_2_ (1L)/GaN(10$\bar 1$0) heterointerface. MoS_2_ (1L) denotes monolayer MoS_2_. The blue part in **b** suggests the charge density distribution at the section. CA charge accumulation, CR charge reduction. Eight Ls in **d** denotes the first 8 layers of GaN. **e** The averaged potential variation along the direction perpendicular to the MoS_2_ (1L)/GaN(10$\bar 1$0) heterointerface

To explain the significantly improved performance of the device, direct calculations of electronic properties of MoS_2_/GaN(10$\bar 1$0) heterointerface at atomic level were conducted. First, to determine the band alignment of GaN(10$\bar 1$0) surface and MoS_2_ (Fig. 4a), the density of states were calculated with the hybrid functional (HSE06) employed. The near-perfect agreement of the calculated electron affinity (3.38 eV) of GaN(10$\bar 1$0) surface with its experimental value, i.e., 3.4 ± 0.1 eV^42^, as well as the outstanding matching of the calculated value (3.71 eV) of MoS_2_ (1L) with the previous GW result, i.e., 3.74 eV^43^, verifies the high accuracy of our simulation methods and models. The band alignment between monolayer and bilayer MoS_2_ and GaN(10$\bar 1$0) surface belongs to type-I. Moreover, taking the detailed band structure calculations for MoS_2_ with different thicknesses from 1L to bulk in Supplementary Figure 11 into consideration, it is found that MoS_2_, regardless of its thickness, can form a straddling gap with GaN(10$\bar 1$0) surface. XPS studies in Supplementary Figure 12 further confirmed that a type-I band alignment was obtained between MoS_*x*_/GaN. The flat band diagram is illustrated in Supplementary Figure 13 based on XPS measurement, indicating that the conduction band offset of MoS_2_/GaN is 0.25 eV. Based on these calculated and experimental results, the bandgap diagram of the entire device under illumination is shown in Supplementary Figure 14. Such straddling band structure facilitates the charge carrier transfer from GaN to MoS_2_. To further study the ability of charge carrier migration from GaN to MoS_2_, the charge density, differential charge density, and density of states of MoS_2_ (1L)/GaN(10$\bar 1$0) heterointerface were calculated. From charge density in Fig. 4b, wavefunction overlap between S atoms and Ga atoms is evidently observed, suggesting the formation of channels for charge transfer from GaN to MoS_2_. Differential charge density analysis in Fig. 4c further illustrates that apparent charge reduction (gray) is found near the S and Ga atoms while charge accumulation (light-magenta) occurs around the middle region of these two kinds of atoms. It indicates that a considerable portion of electrons are shared by those two kinds of atoms, resulting in new electronic states in this heterointerface. And these induced electronic states were confirmed by its density of states (Fig. 4d). Compared to Fig. 4a, new hybridized states contributed mainly by Ga *d*_*z2*_*, d*_*xz*_*, d*_*yz*_ orbitals and S *p*_*z*_ orbitals are discovered in the MoS_2_/GaN(10$\bar 1$0) vertical heterointerface. These out-of-plane orbitals hybridize and connect the Ga atoms and S atoms in MoS_2_/GaN interface, building a network for itinerant electrons. The electrostatic potential distribution was further calculated to determine the energy barrier between MoS_2_ and GaN. Significantly, the corresponding averaged potential variation along the direction perpendicular to the interface in Fig. 4e shows that there is no energy barrier between MoS_2_ and GaN; and the potential of S atoms is much lower than that of Ga and N, indicating that electrons in GaN are prone to move to MoS_2_. It demonstrates an ideal electron-migration channel for highly efficient charge carrier extraction, due to the suitable electronic properties of the MoS_*x*_@GaN NWs/Si heterostructure, as well as the excellent geometric-matching structure as indicated above. Meanwhile, the atomic hydrogen-free energy of MoS_2_ was also calculated and shown in Supplementary Figure 15, which is very low (0.036 eV), and even comparable to that of Pt (−0.09 eV)^44^, indicating that MoS_*x*_ is intrinsically active for hydrogen evolution reaction, which is another important contributing factor for the high performance.

### The function of GaN nanowire

**Fig. 5:**
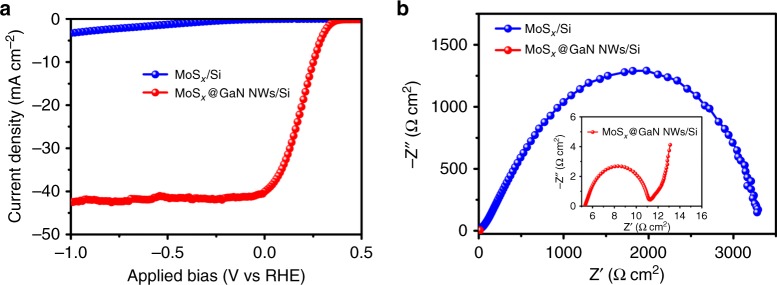
The function of GaN nanowire. **a** *J*–*E* curves of MoS_*x*_/Si and MoS_*x*_@GaN NWs/Si in 0.5 M H_2_SO_4_ under standard one-sun illumination. **b** Electrochemical impedance spectroscopy (EIS) analysis of MoS_*x*_/Si and MoS_*x*_@GaN NWs/ Si. Inset graph is the magnification of EIS of MoS_*x*_@GaN NWs/Si

Control experiments were carried out to better understand the function of GaN nanowire. The activity of MoS_*x*_/Si and MoS_*x*_@GaN NWs/Si was examined (Fig. 5a). It is observed that MoS_*x*_/Si, holding a planar structure like the bare Si substrate (Supplementary Figure 16), exhibits much lower activity than MoS_*x*_@GaN NWs/Si. In detail, the onset potential of MoS_*x*_/Si is ~−0.12 V. As compared to MoS_*x*_@GaN NWs/Si, the current density of MoS_*x*_/Si is also much smaller. To gain more insights into the interfacial properties of MoS_*x*_/Si and MoS_*x*_@GaN NWs/Si, electrochemical impedance spectroscopy (EIS) measurements under standard one-sun illumination were performed. As shown in Fig. 5b, the charge transfer resistance of MoS_*x*_@GaN NWs/Si was much lower than that of MoS_*x*_/Si. These experimental results, in excellent accordance with the calculated structural and electronic properties of MoS_2_/GaN, provide unambiguous evidence that GaN nanowire is an outstanding linker between MoS_*x*_ and planar silicon, endowing the device with ideal electron-migration channel for highly efficient solar-to-hydrogen conversion.

## Discussion

In summary, we have demonstrated that GaN nanowire can function as an ideal linker between planar Si and MoS_*x*_ to realize a unique high-quality shell-core heterostructure of MoS_*x*_@GaN NWs/Si for PEC water splitting. The integrated photocathode is noble-metal-free and is capable of exposing high-density active sites and harvesting solar light effectively. Most importantly, the unique electronic interaction, as well as the superior geometric-matching structure between GaN and MoS_*x*_, offers an ideal electron-migration channel for high charge carrier extraction efficiency. These synergetic effects result in extraordinary performance. An impressive photocurrent density of 40 ± 1 mA cm^−2^ at 0 V vs. RHE, a large onset potential of +0.4 V, and a high level of stability are obtained under standard one-sun illumination. It is important to notice that the materials being used in the photoelectrode, including GaN and Si, are industry-ready, and the photoelectrode fabrication process involves the use of highly controllable industry-scale manufacturing process. As such, the presented outstanding photocathode can be reproducibly fabricated on a large scale, providing a promising route for the practical conversion and storage of solar energy into hydrogen.

## Methods

### Growth of GaN nanowires on planar n^+^–p junction silicon

A standard thermal diffusion process was first employed for preparing planar n^+^–p junction silicon. The orientation of the silicon wafer is (100). GaN nanowire arrays were then grown on planar n^+^–p junction silicon by plasma-assisted molecular beam epitaxy at 750 °C with a desired time under nitrogen-rich conditions. The nitrogen flow rate was 1.0 standard cubic centimeter per minute (sccm). And the forward plasma power and Ga flux were 350 W and ~ 8 × 10^−8^ Torr, respectively. There is no AlN buffer layer utilized during the MBE growth.

### Electrodeposition of MoS_*x*_ on GaN NWs/Si

MoS_*x*_@GaN NWs/Si was prepared by a facile electrodeposition method with a minor modification compared to previous report^37^. The electrode of GaN NWs/Si was immersed into an aqueous solution of (NH_4_)_2_MoS_4_ with a desired concentration. Desired cycles of cyclic voltammetry were conducted in a PEC chamber using a three-electrode configuration. The scan rate was 100 mV s^−1^. After the deposition, the electrode was washed with distilled water several times. For comparison, the same protocol was used for depositing MoS_*x*_ on bare n^+^−p junction Si without GaN NWs.

### The calculation of the loading density of MoS_*x*_

MoS_2_ was obtained from the reduction of (NH_4_)_2_MoS_4_ by the electrons as the following equation: MoS_4_^2−^ + 2H_2_O + 2e^−^ = MoS_2_ + 2HS^−^ + 2HO^−^. Therefore, the loading density of MoS_*x*_ = (Moles of the consumed electrons)/(2 × surface area of the sample).

### Characterization of the electrodes

TEM and SEM images were recorded on an FEI Tecnai G2 F20 microscope and an Inspect F-50 FE-SEM system, respectively. XPS was performed using a Thermo Scientific K-Alpha XPS system with a monochromatic Al Kα source (*hν* = 1486.6 eV). Charging effects were compensated by a flood gun. XRD patterns were determined on a Bruker D8 Discovery X-ray diffractometer using Cu-Kα radiation. A Cary 5000 UV–Vis–NIR spectrophotometer was utilized for measuring the UV–Vis reflectance spectra. The baseline was collected using a mirror accessory.

### Photoelectrochemical reactions

The photoelectrochemical reactions were conducted in a typical three-electrode cell. Pt wire and Ag/AgCl were utilized as the counter electrode and reference electrode, respectively. 200 mL of 0.5 mol/L H_2_SO_4_ aqueous solution was used as the electrolyte. A solar simulator (Oriel LCS-100) was used as the light source. The light intensity approaching the surface of the sample is calibrated to be 100 mW cm^−2^. The photoelectrocatalysis data were recorded using an Interface 1000E potentiostat (Gamry Instruments). A small fraction of headspace products in the chamber was analyzed by a gas chromatography (GC-8A, Shimadzu).

### Density functional theory calculation

Density functional theory calculations were performed using the generalized gradient approximation for the exchange-correlation potential, the projector augmented wave method^45^, and a plane-wave basis set as implemented in the Vienna ab initio simulation package^46^. The energy cutoff for the plane-wave basis was set to 500 eV for all calculations only concerned with MoS_2_ and 550 eV for those referring to GaN. Two k-meshes of 13 × 13 × 1 and 13 × 9 × 1 were adopted to primitive cells of MoS_2_ and GaN(10$\bar 1$0)-wurtzite, separately, and the mesh density of k points was kept fixed when performing calculations related with their respective supercells. In optimizing the system geometry, van der Waals (vdW) interactions were considered by the vdW-DF level with the optB86 exchange functional (optB86-vdW)^47^. And all structures were fully relaxed until the net force per atom was less than 0.01 eV Å^−1^. All electronic properties of MoS_2_/GaN(10$\bar 1$0) heterointerface were predicted with the hybrid functional (HSE06)^48^. With respect to the geometry structure of MoS_2_/GaN(10$\bar 1$0) heterointerface, 32 layers of Ga and N atoms were used, and two different kinds of pseudo hydrogen atoms were employed to passivate the dangling bonds at the bottom of the GaN (wurtzite) slab. Density functional perturbation theory (DFPT)^49^ was employed to calculate entropy and zero point energies. Regarding DFPT calculations, the energy cutoff for the plane-wave basis was set to 600 eV, and corresponding mesh density of k points was nearly doubled while structures were fully relaxed until the residual force per atom was less than 0.0001 eV Å^−1^.

### Free energy calculation

The free energies of the hydrogen-adsorbed state are calculated by both Eqs. (1) and (2), as following:

1 $$\Delta G_{\mathrm{H} \ast } = E_{{\it{ad}}}\left( {\mathrm{H}} \right) + \Delta E_{{{ZPE}}} - {{T}}\Delta {S}_{\mathrm{H}}$$

2 $$E_{{\it{ad}}}\left( {\mathrm{H}} \right) = \frac{1}{n}\left( {E_{{{\mathrm{surface}} + n{\mathrm{H}}}} - {{E}}_{{\mathrm{surface}}} - \frac{n}{2}E_{{\mathrm{H}}_2}} \right)$$

where H^*^ means a hydrogen atom adsorbed on the surface. *E*_ad_(H) is the hydrogen adsorption energy, and *n* is the number of H atoms. *E*_surface+*n*H_ is the total energy of the fully relaxed adsorption configurations, and *E*_H2_ is the total energy of H_2_ in the gas phase while *E*_surface_ is obtained from a clean surface without H atoms being adsorbed. ∆*E*_ZPE_ and *T*∆*S*_H_ denote the difference between the adsorbed and the gas phase of hydrogen in zero point energy and entropy energy, respectively. Since the vibrational entropy for H^*^ is quite small, for all the free energy calculations, we take the following Eq. (3) as an approximation:

3 $$T\Delta S_{\rm{H}} \cong - \frac{1}{2}{\it{T}}S_{{\rm{H}}_2}$$

where $S_{{\rm{H}}_2}$is the entropy of H_2_ in the gas phase at standard conditions.

## Electronic supplementary material

Supplementary Information

## Data Availability

The data that support the plots within this paper and other findings of this study are available from the corresponding author on reasonable request.
