# Supplementary material for: Gallium nitride nanowire as a linker of molybdenum sulfides and silicon for photoelectrocatalytic water splitting
Source: Nat Commun. 2018 Sep 21;9:3856. doi: 10.1038/s41467-018-06140-1 (PMC6155116; doi:10.1038/s41467-018-06140-1)
Supplement: Supplementary file 1 — Supplementary Information [file 41467_2018_6140_MOESM1_ESM.pdf]

Supplementary information

Gallium nitride nanowire as a linker of molybdenum sulfides and silicon for photoelectrocatalytic water splitting

Zhou et al.

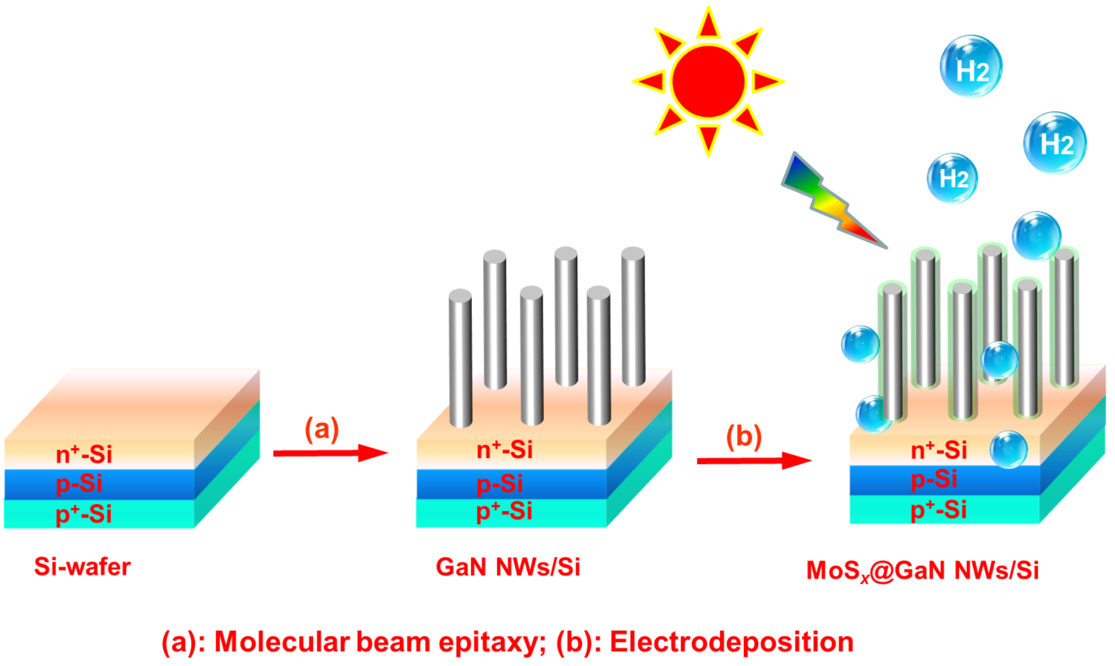

Supplementary Figure 1 | Fabrication of shell-core MoS_x@GaN NWs/Si by molecular beam epitaxy and electrodeposition for solar water splitting to hydrogen.

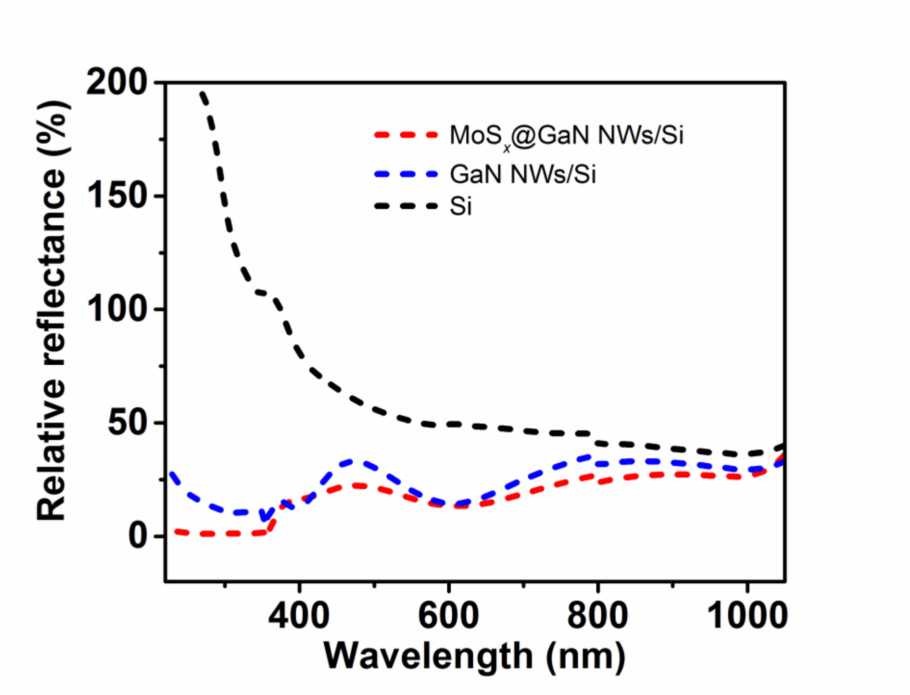

Supplementary Figure 2 | UV-Vis relative reflectance spectra of Si, GaN NWs/Si, and MoS_x@GaN NWs/Si measured under identical conditions.

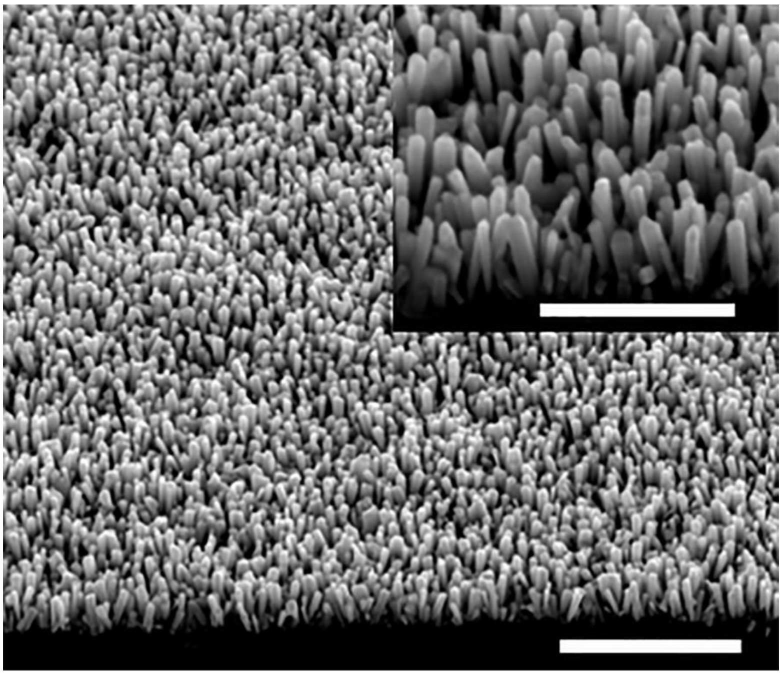

Supplementary Figure 3 | SEM image of GaN NWs/Si in the absence of MoS_x. Scale bar: 1 μm, inset 500 nm.

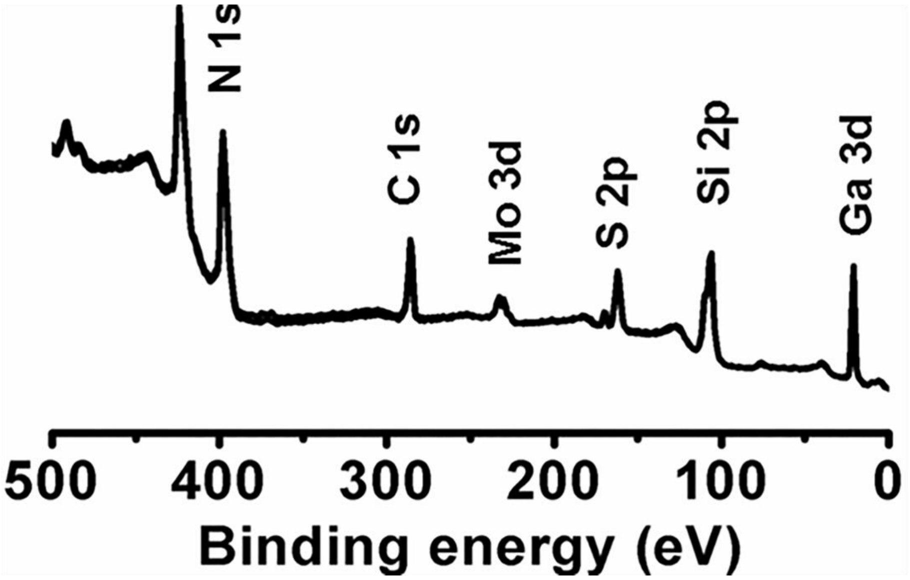

Supplementary Figure 4 | X-ray photoelectron spectrum of MoS_x@GaN NWs/Si.

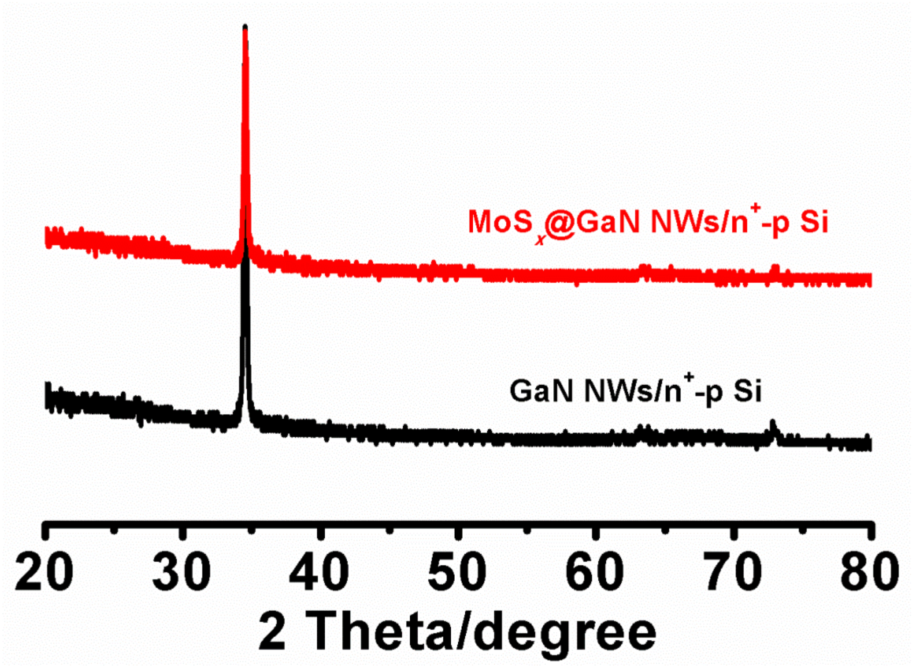

Supplementary Figure 5 | X-ray diffraction spectral of GaN NWs/Si and MoS_x@GaN NWs/Si.

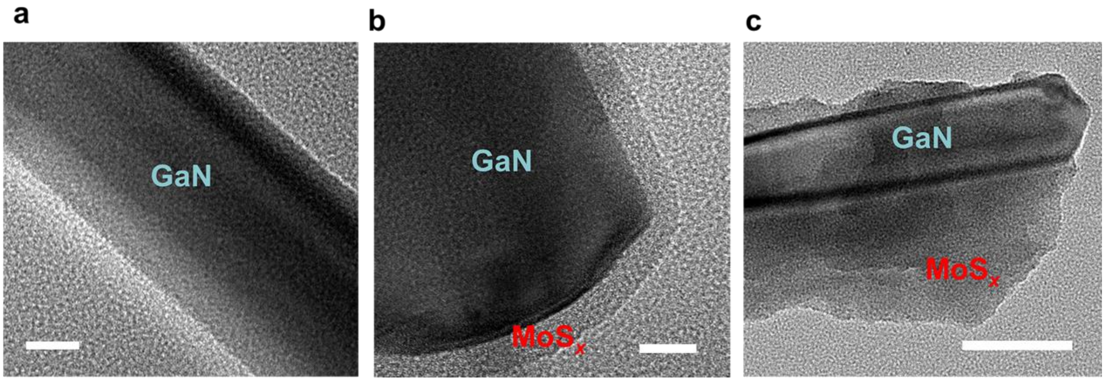

Supplementary Figure 6 | TEM images of GaN NWs/Si deposited with various densities of MoS_x.

a 0 μmol cm^{-2}, **b** 0.73μmol cm^{-2}, and **c** 1.72μmol cm^{-2}. Scale bars: **a-b** 10 nm and **c** 50 nm

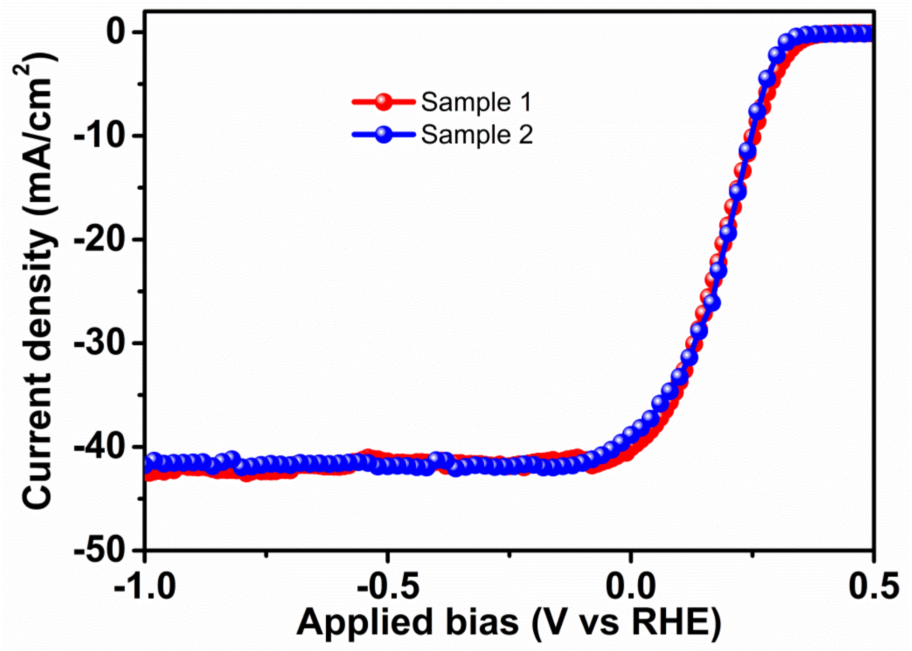

Supplementary Figure 7 | The variation of PEC performance of MoS_x@GaN NWs/Si with 0.73 μmol cm^{-2} of MoS_x under standard one-sun illumination in 0.5 M H_2SO_4.

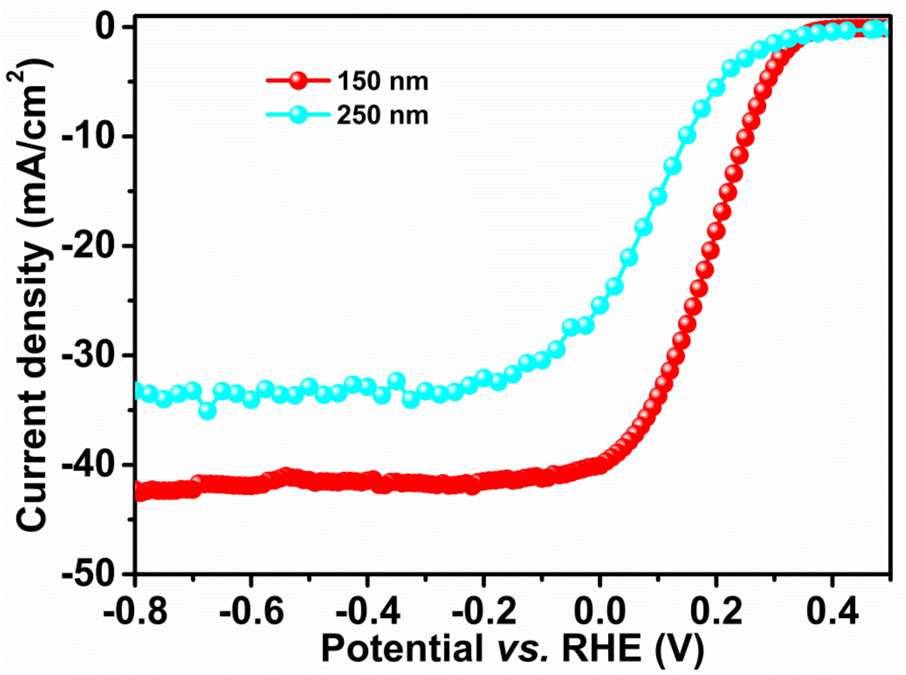

Supplementary Figure 8 | PEC performance of MoS_x@GaN NWs/Si with different lengths of GaN NWs.

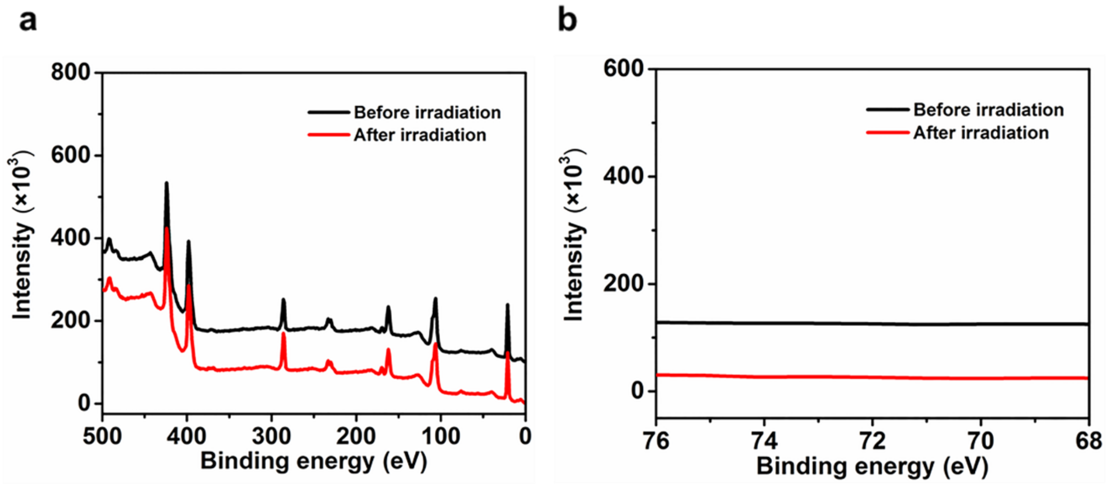

Supplementary Figure 9 | X-ray photoelectron spectrum. **a** MoS_x@GaN NWs/Si and **b** the core level spectra of Pt 4f before and after 10 hours reaction under standard one-sun irradiation in 0.5 mol L^{-1} H_2SO_4 aqueous solution.

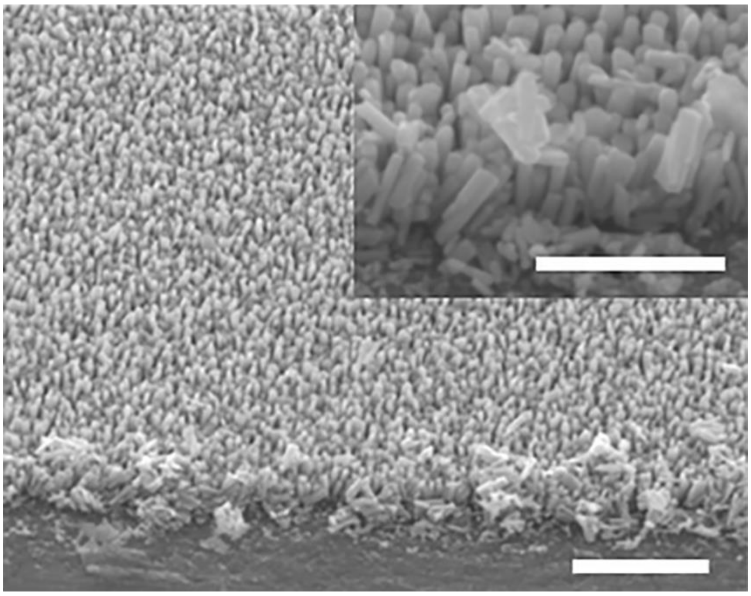

Supplementary Figure 10 | SEM image of MoS_x@GaN NWs/Si after 10 hours reaction under standard one-sun irradiation in 0.5 mol L^{-1} H_2SO_4 aqueous solution. Scale bar: 2 μm, inset 500 nm.

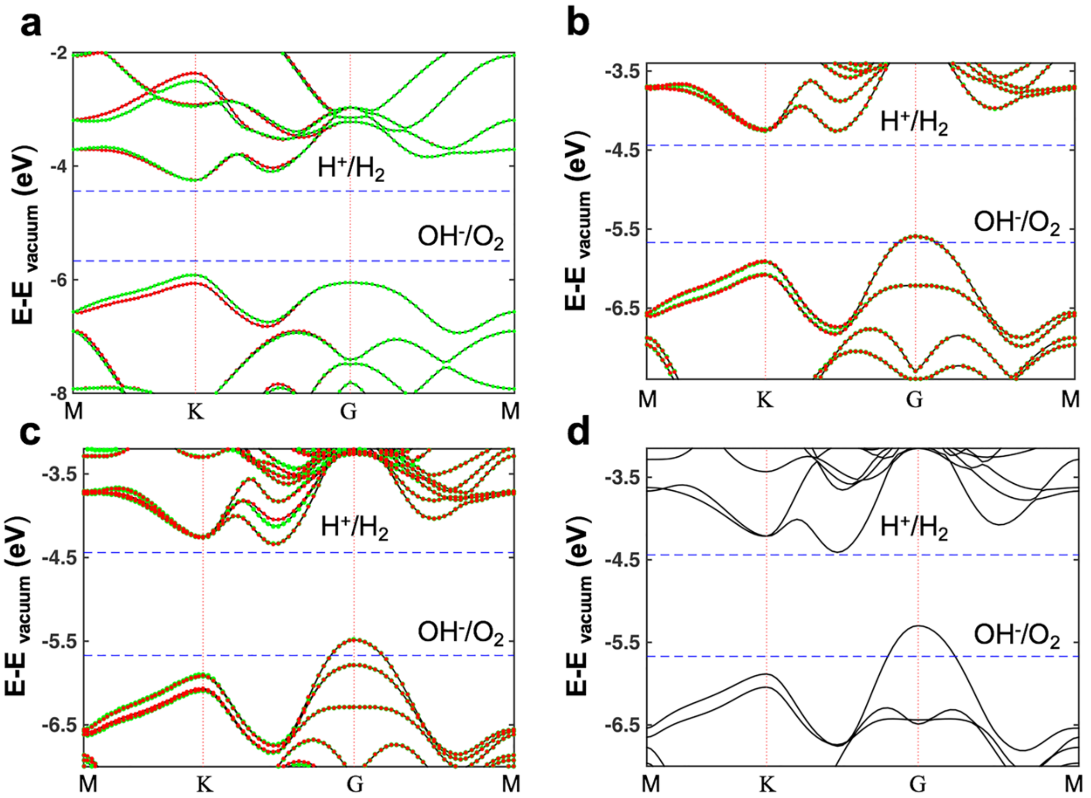

Supplementary Figure 11 | Band structure of MoS_2 with different thicknesses. **a** MoS_2-1L, **b** MoS_2-2L, **c** MoS_2-3L, **d** MoS_2-bulk. Spin-orbital coupling is taken into consideration for band structure calculations of few-layer MoS_2. In even-layer and bulk MoS_2, their inversion symmetry combined with time reversal leads to Kramers degeneracy, which means that no spin splitting exists. Oxidation-Reduction-Potential (ORP) is marked as OH^-/O_2 and H^+/H_2 with blue dashed line. This figure shows that regardless of the thickness of MoS_2, its conduction band minimum (CBM) remains at an optimal position referring to the reduction potential of H_2O splitting.

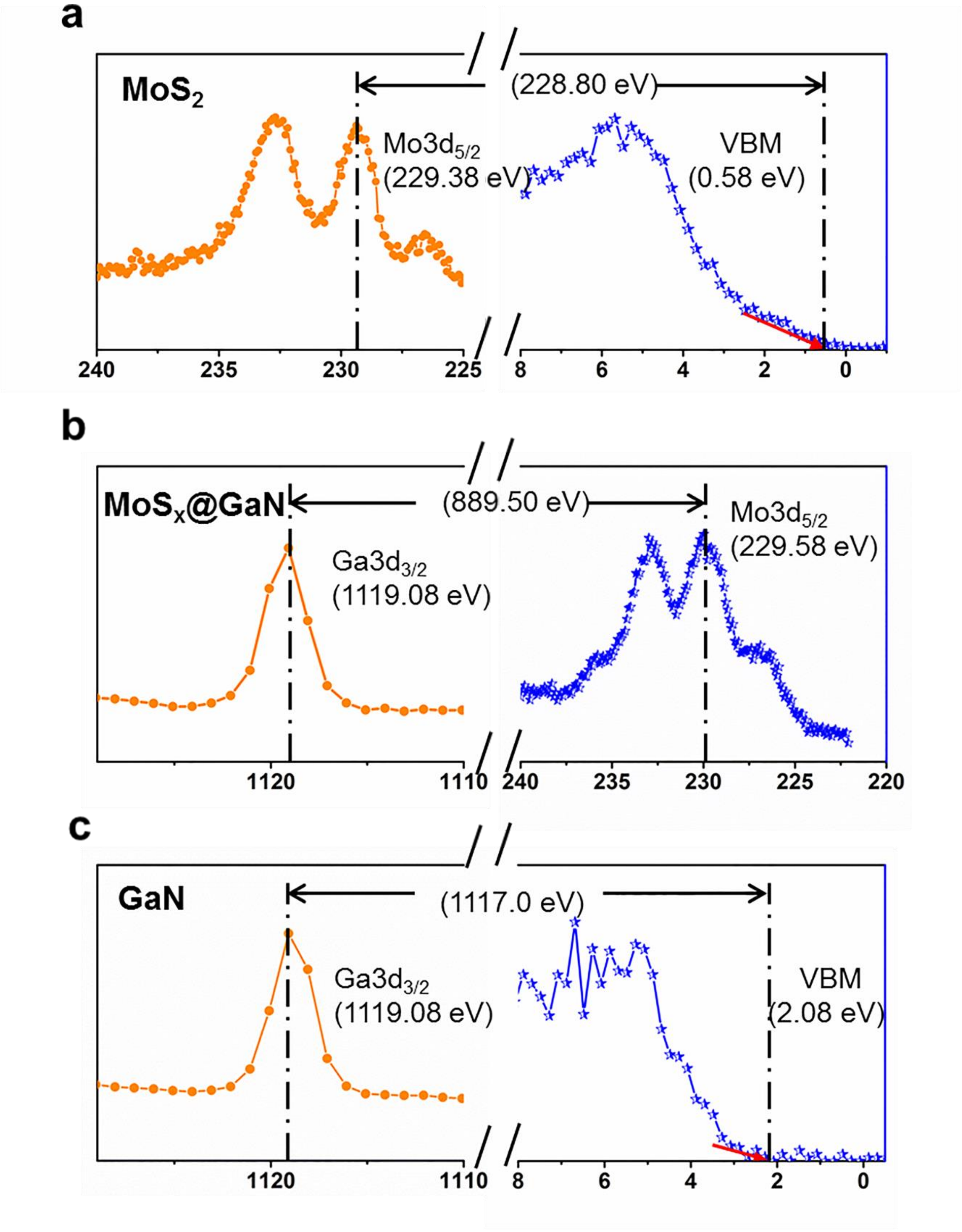

Supplementary Figure 12 | Core-level and valence band spectra based on XPS measurement. **a** MoS_2, **b** MoS_x@GaN, and **c** GaN.

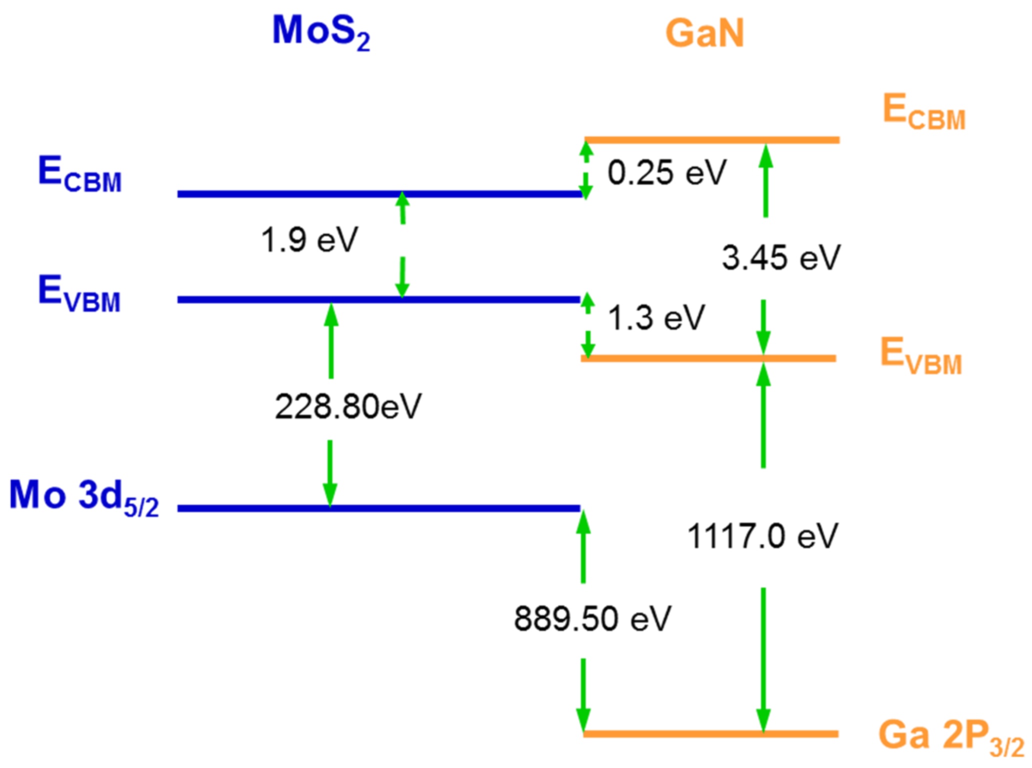

Supplementary Figure 13 | The flat band alignment diagram of MoS_x@GaN based on XPS measurement.

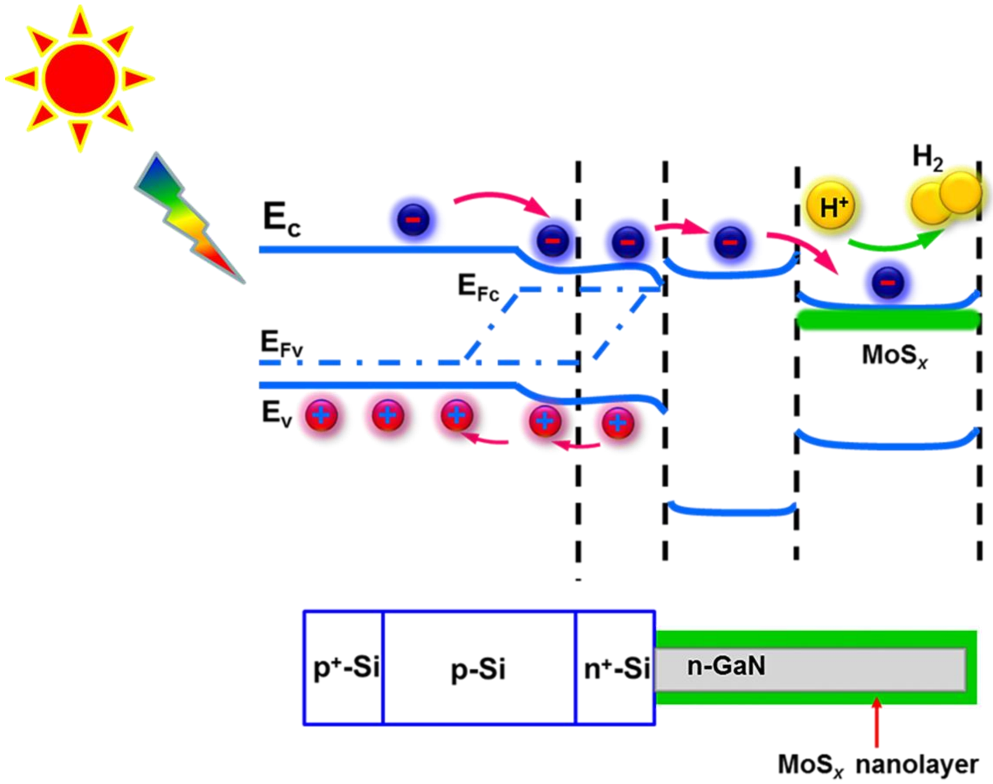

Supplementary Figure 14 | Energy band diagram of MoS_x@GaN NWs/Si under illumination. Herein, both Si and GaN are heavily n-type doped, which can facilitate the electrons transfer from Si to GaN. Moreover, under irradiation, the abundant photogenerated electrons result in flat-band condition, which decreases the upward bending of surface energy and promotes the electron injection from n^+-Si player to n-GaN at a small applied bias. The unique electronic interaction and outstanding geometric-matching structure between GaN and MoS_2 further provides an ideal electron-migration channel for the electrons transfer to HER catalytic sites.

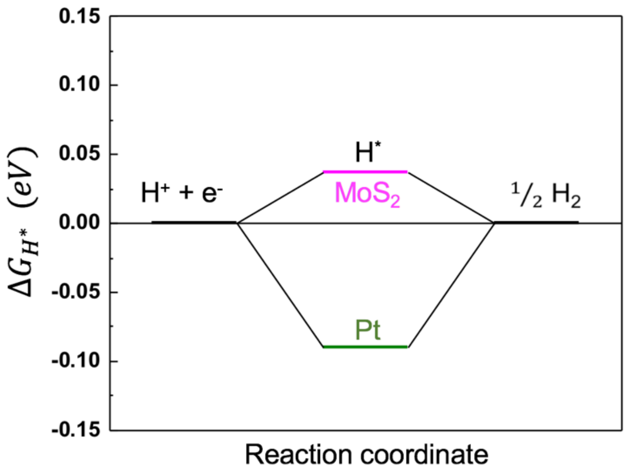

Supplementary Figure 15 | Free energy diagram of MoS_2 for HER at equilibrium (U=0) relative to SHE at pH = 0.

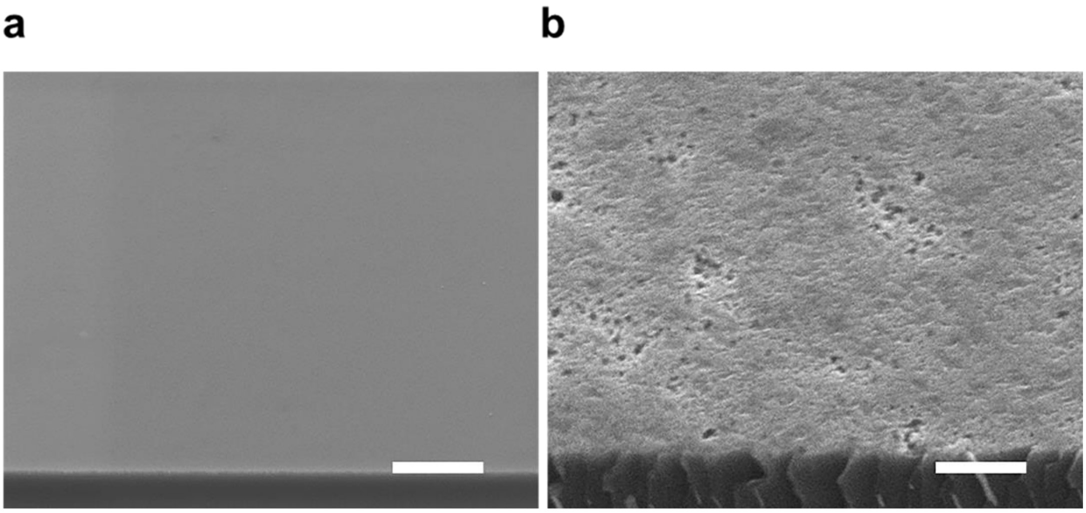

Supplementary Figure 16 | 45°**-** titled SEM images. **a** bare planar n^{+}**-**p junction Si and **b** MoS_x/Si.

Scale bar: 1 μm.
